# Predicting diffusion-FLAIR mismatch from B1000 and ADC without FLAIR: A deep learning-based approach

**DOI:** 10.1038/s41598-026-55388-x

**Published:** 2026-06-02

**Authors:** Pum Jun Kim, Dongyoung Kim, Joonwon Lee, Hyung Chan Kim, Jung Hwa Seo, Sukyoon Lee, Doo Hyuk Kwon, Hyungjong Park, Chang Hun Kim, Ho-Joon Lee, Yeonah Kang, Jaejun Yoo, Seongho Park

**Affiliations:** 1 Graduate School of Artificial Intelligence, Ulsan National Institute of Science and Technology, Ulsan, South Korea; 2 Department of Neurology, Inje University Haeundae Paik Hospital, Busan, South Korea; 3 Department of Neurology, Ulsan Hospital, Ulsan, South Korea; 4 Department of Neurology, Dong-A University College of Medicine, Busan, South Korea; 5 Department of Neurology, Busan Paik Hospital, Inje University College of Medicine, Busan, South Korea; 6 Department of Neurology, Yeungnam University College of Medicine, Daegu, South Korea; 7 Department of Neurology, Kyungpook National University Hospital, School of Medicine, Kyungpook National University, Daegu, South Korea; 8 Department of Neurology, Gyeongsang National University Hospital, Jinju, South Korea; 9 Department of Radiology, Haeundae Paik Hospital, Inje University, Busan, South Korea; 10 Seoil Medical Group Clinic, Busan, South Korea; 11 Department of Neurology, College of Medicine, Hanyang University Guri Hospital, Hanyang University, Guri, South Korea; 12 Department of Neurology, Kyung Hee University Graduate School, Seoul, South Korea

**Keywords:** Diffusion-FLAIR mismatch, Acute ischemic stroke, Deep learning, Apparent diffusion coefficient, B1000, Biomarkers, Computational biology and bioinformatics, Medical research, Neurology

## Abstract

Diffusion-FLAIR mismatch (DFM), defined as the discrepancy between diffusion-weighted imaging (DWI) and FLAIR sequences, is a key imaging biomarker used to identify patients with unclear symptom onset or those likely to benefit from recanalization therapy. However, FLAIR imaging is often limited in acute clinical settings due to time constraints, patient instability, or scanner availability. Therefore, alternative approaches to assess DFM without relying on FLAIR are needed. This study aimed to develop a deep learning model that predicts DFM using only B1000 and apparent diffusion coefficient (ADC) images, and to evaluate its performance. This study was conducted using multicenter stroke registry-based cohorts. After excluding cases not suitable for analysis, 2,369 cases were included in the derivation cohort for model development, and 679 cases from two independent stroke centers were included as external validation cohorts. Model performance was assessed based on two binary classification schemes. The Broad classification included all non-match cases, comprising both clearly defined mismatches and ambiguous cases with subtle or focal FLAIR changes, while the Focused classification excluded such ambiguous cases, including only clearly defined match and mismatch instances. In the Focused classification setting, the AI model achieved an AUROC of 0.92 (95% CI: 0.88–0.95) on the external validation dataset, significantly outperforming the average AUROC of human experts, which was 0.82 (95% CI: 0.77–0.87) (*p* < 0.001, AUROC difference 0.10). Overall, the Focused classification yielded higher performance than the Broad classification, and this trend was consistent for both the AI model and human readers. We developed a deep learning-based classifier capable of predicting DFM using only B1000 and ADC images, without requiring FLAIR. The model outperformed human experts and demonstrates potential as a complementary diagnostic tool in acute stroke settings where FLAIR imaging is unavailable, supporting its future integration into decision-support systems.

**Supplementary Information:**

The online version contains supplementary material available at 10.1038/s41598-026-55388-x.

## Introduction

Diffusion-FLAIR mismatch (DFM), defined as the discrepancy between diffusion-weighted imaging (DWI) and fluid-attenuated inversion recovery (FLAIR), is a critical imaging biomarker used to determine the eligibility for intravenous thrombolysis (tPA) in patients with acute ischemic stroke of unknown onset^1–5^.Recent randomized controlled trials involving endovascular therapy (EVT) have also utilized DFM as a method to indicate the presence of salvageable brain tissue^6,7^.

However, the assessment of DFM remains limited in real-world clinical practice. DFM evaluation requires both DWI and FLAIR sequences, but FLAIR acquisition may not always be reliably available or diagnostically usable in acute stroke workflows, particularly in time-critical or unstable patients^8,9^. In real-world settings, FLAIR sequences can be degraded by motion artifacts, omitted in abbreviated protocols, or rendered non-diagnostic due to limited patient cooperation. As a result, DFM assessment may become impractical or delayed in a subset of patients, especially when FLAIR images are unavailable or of insufficient quality^10^.

Therefore, there is a need for approaches capable of assessing DFM without strict reliance on FLAIR imaging. Such a method would improve the robustness and continuity of clinical decision-making by enabling appropriate diagnostic and treatment decisions even when FLAIR is unavailable, unreliable, or intentionally omitted as part of an abbreviated protocol.

Meanwhile, the apparent diffusion coefficient (ADC), which is acquired alongside DWI, provides quantitative information on diffusion restriction in brain tissue^11^. Together with the B1000 sequence, ADC images are included in most routine MRI protocols, offering high accessibility. B1000 images reflect diffusion restriction but also include T2-related signal components. It is therefore conceivable that the relative signal characteristics between B1000 and ADC may encode latent information regarding tissue states associated with T2 signal increase, such as edema-related signal changes accompanying diffusion restriction. For example, even among lesions with comparable DWI hyperintensity, the degree of ADC reduction, the relative magnitude of B1000 signal, and the internal texture or spatial heterogeneity of the lesion may differ between tissue states with and without accompanying FLAIR hyperintensity; such multidimensional signal combinations are difficult for human readers to integrate intuitively as a single threshold-based rule, but can potentially be captured by deep-learning models. Indeed, the potential informational overlap between DWI-derived sequences and FLAIR has been suggested in prior studies that explored the relative combination of DWI and ADC as a surrogate marker for aspects of DWI–FLAIR mismatch^12^or as an indicator related to lesion fate and penumbra^13^. However, these approaches have largely relied on predefined thresholds or summary metrics, and the underlying signal relationships are often subtle and difficult for human observers to evaluate intuitively. Consequently, the clinical utility of simple rule-based approaches remains limited.

Deep learning–based analysis offers significant potential to overcome this limitation, as it can learn and recognize complex imaging patterns that may be difficult for the human eye to interpret^14^. Such technology may serve as a novel diagnostic tool by complementing or enhancing traditional radiologic assessment.

Accordingly, this study aimed to develop and validate a deep learning–based classifier capable of predicting DFM using only B1000 and ADC images, and to evaluate its performance in comparison with human experts.

Methods.

## Study design and data source

This study used retrospective data from four previously established stroke cohorts derived from a prospective stroke registry. Two cohorts from separate hospitals were used for model development (derivation), while external validation was performed using datasets from two independent stroke centers, referred to as the Ext_A and Ext_B cohorts in this study. The design and patient selection criteria for these datasets have been described in detail in a previous publication^15^. In brief, patients aged ≥ 18 years with acute ischemic stroke who underwent MRI—including FLAIR, DWI (b = 0 and b = 1000 s/mm²), and apparent diffusion coefficient (ADC) sequences—during a single imaging session were included. Only patients with diffusion-restricted lesions larger than 5 mL were selected, based on an automated segmentation tool developed in prior work^16^. Lesions smaller than 5 mL were excluded because they are rarely relevant to reperfusion decision-making and often present as multiple scattered, dot-like infarcts, for which reliable diffusion–FLAIR mismatch labeling is inherently difficult. This exclusion was applied to ensure robust ground truth construction and to align model training with clinically meaningful and safe application.

This study was approved by the institutional review boards of all participating centers, with written informed consent waived due to the retrospective nature of the study. All data were anonymized according to institutional protocols. All methods were carried out in accordance with relevant guidelines and regulations, including the Declaration of Helsinki. The study followed STARD reporting guidelines^17^.

## Ground truth annotation process for the datasets

The ground truth labeling process used in this study followed a previously established protocol described in detail in our earlier work^15^. In brief, five stroke experts (four neurointerventionists and one stroke neurologist) independently labeled DFM status by assessing the signal discrepancies between DWI and FLAIR images obtained from the same session. Lesions were classified into five categories: (a) DWI-FLAIR match, (b) mismatch, (c) subtle FLAIR change, (d) regional heterogeneous FLAIR change, and (e) non-ischemic or unanalyzable cases; representative examples and per-category descriptors are provided in Figure S1. For the purposes of this study, categories (a) and (b) were operationally regarded as clear cases—those in which the consensus review could establish a confident determination of either the presence or absence of FLAIR hyperintensity spatially corresponding to the DWI lesion. In contrast, categories (c) and (d) were regarded as ambiguous cases, in which faint, equivocal, or spatially heterogeneous FLAIR changes introduced uncertainty in assigning a definitive match or mismatch label.

For analysis, two binary classification schemes were constructed based on categories (a) through (d). The Broad classification (Label Set I) was designed to approximate a more inclusive clinical setting in which both clearly defined and ambiguous cases are encountered; therefore, categories (b), (c), and (d) were grouped as “non-match” and compared against category (a) (DF match). In contrast, the Focused classification (Label Set II) was designed to evaluate model and reader performance under a cleaner labeling condition by including only clearly defined match (a) and mismatch (b) cases and excluding the ambiguous categories (c) and (d). Comparing the two schemes therefore allowed us to assess how label ambiguity affected the performance of both the AI model and human readers.

To ensure consistency and reliability, the labeling process included consensus review steps and inter-rater agreement assessments. Interrater agreement was assessed on a predefined subset of cases that were independently reviewed by all five raters prior to dataset partitioning. Following iterative consensus review, Fleiss’ Kappa for this subset reached 0.91. After consensus was established, the remaining cases were independently labeled by individual raters according to the agreed criteria. Reviewers were blinded to clinical data and were provided only with imaging. The same labeling structure was applied to both the derivation and external validation cohorts.

## Reader study dataset

To compare the DFM classification performance of the developed AI model with that of human experts, a reader study was conducted using the external validation cohort. The reader group consisted of two board-certified neuroradiologists and one neurointerventionist, each with more than 10 years of experience in brain imaging or neurointervention. These readers were independent from both the institutions that provided the external datasets and the five stroke experts who participated in the ground truth annotation process.

Reader training was conducted using 150 cases randomly selected from the model development dataset. Each case included the reference label along with B1000, ADC, and FLAIR images, as well as infarct mask overlays. FLAIR images were co-registered to the DWI sequence to allow readers to become familiar with the lesion labeling criteria and to understand the correspondence between sequences.

After the training phase, readers were presented with the external validation dataset for actual evaluation. During this evaluation, readers assessed only B1000 and ADC images, without access to FLAIR images or clinical information, mirroring the imaging input available to the AI model and minimizing potential bias. Additional supporting images, including multiple ADC window settings, were provided to facilitate detailed assessment (see Figure S2).

Reader assessments of DFM were recorded on a three-level confidence scale, which was then converted into a likelihood of DF match (LOD) score. The LOD score ranged from 0 to 1, where 0 indicated DF mismatch, 0.5 indicated uncertain mismatch, and 1 indicated DF match.

### Data preprocessing and model development

The data preprocessing pipeline and model architecture used in this study were based on methods previously established in our earlier publication^15^. In brief, all MRI sequences—including B1000, ADC, and FLAIR—underwent a standardized preprocessing protocol involving image normalization and interpolation to address differences in resolution across sequences. Each image was resized to 256 × 256 pixels, and the number of axial slices was standardized to 36 using nearest-neighbor interpolation. Image-wise normalization was applied to scale pixel values between 0 and 1.

Since B1000 and ADC images are inherently co-registered, no further alignment was necessary. However, FLAIR images were registered to the DWI space using the TRSAA method, which demonstrated superior performance compared to other registration techniques such as Affine, SyNRA, Elastic, and ElasticSyN, as implemented in the ANTsPy library (version 0.3.8).

A binary classification model was constructed using a DenseNet-based 3D convolutional neural network (3D CNN) consisting of four repeated dense blocks and transition layers. The input consisted of co-registered B1000 and ADC volumes. Each dense block contained 1 × 1 × 1 and 3 × 3 × 3 convolutional layers, with respective block depths of 6, 12, 24, and 16. The final linear layer included two nodes for predicting match vs. mismatch. Batch normalization and 3D convolution were used throughout the network, with stride 2 applied to all convolutional and pooling layers outside the dense blocks and transition layers.

During development, 90% of the derivation dataset was used for training and hyperparameter tuning, while 10% was held out for internal evaluation. The model was implemented using Python (version 3.8.17) and PyTorch (version 1.13.1 + cu117).

## Algorithm validation and statistical analysis

The performance of the developed model was evaluated on both the derivation and external validation datasets. For the external validation cohort, we conducted a direct comparison between the AI model and human experts. To effectively compare model performance with that of multiple readers, the average of the Likelihood of DF match (LOD) scores from the three readers was used as the representative human score.

For the derivation dataset, model performance was evaluated using a cross-validation strategy as illustrated in Figure S3. For external validation, the entire dataset from each institution was used as a test set. Discriminative performance was assessed using the area under the receiver operating characteristic curve (AUROC) along with 95% confidence intervals (CIs). Differences in AUROC values between groups were tested using the DeLong test^18^. Additional performance metrics included the positive predictive value (PPV) and negative predictive value (NPV). Sensitivity and specificity were calculated at the optimal threshold defined by the Youden Index (J = sensitivity + specificity – 1).

Subgroup analyses were performed to evaluate model generalizability under varying clinical conditions, including stroke onset time, infarct core volume, cohort site, and label scheme (broad vs. focused classification). For subgroup analyses, the AUROC of the AI model was compared with the average AUROC of the three human readers. For infarct core volume subgroups, AUROC differences were compared using DeLong’s test within each subgroup. For stroke onset-to-imaging time subgroups, which involved multiple time intervals, differences in AUROC between the AI model and the readers’ average across intervals were assessed using a weighted chi-square test, with weights inversely proportional to the variance of each AUROC estimate.

To assess the model’s calibration and overall predictive reliability, the Brier score was computed and calibration curves were generated^19,20^. The Brier score quantifies the accuracy of probabilistic predictions, and the calibration curve visualizes the agreement between predicted probabilities and observed outcomes.

Prediction accuracy was further evaluated by comparing the AI and human expert results using contingency tables, applied to the same external validation cases assessed by both. For this comparison, the AI probability scores—obtained from the model’s two-node softmax output as continuous match probabilities—were binarized using the predefined Youden-index cutoffs derived from the derivation cohort, and human classifications were determined by majority voting (> 50%) among the three readers. Statistical comparisons were made using chi-square and McNemar’s tests to assess performance differences and agreement levels.

To enhance interpretability of the image-based model, saliency maps were generated using Guided Grad-CAM^21^. This technique combines gradient-based localization with class activation mapping to highlight image regions that most strongly contributed to the model’s prediction.

Inter-reader agreement among the three human evaluators was assessed using Fleiss’ kappa statistic.

All statistical analyses were performed using R software (version 4.1.3), and p-values < 0.05 were considered statistically significant for two-sided tests.

## Results

A total of 3,022 patients from the derivation cohorts and 753 patients from the external validation cohorts (Ext_A and Ext_B) initially considered for analysis (Figure S4). After excluding cases not suitable for analysis, 2,369 patients from the derivation cohorts and 679 patients from the external validation cohorts were included in the final analytic dataset. The external validation dataset consisted of 350 patients from Ext_A and 329 patients from Ext_B. Among the included patients, 1,443 (60.9%) in the derivation cohort, 213 (60.8%) in Ext_A, and 160 (48.6%) in Ext_B were labeled as diffusion-FLAIR match. The Ext_B cohort showed a higher proportion of mismatch cases compared to the others. Cases categorized as having ambiguous FLAIR changes (i.e., subtle or regional heterogeneous changes) accounted for 699 (29.5%) in the derivation cohort, 86 (24.6%) in Ext_A, and 123 (37.3%) in Ext_B (Table 1).

**Table 1:** Cohort Characteristics and Label Distribution.

Age in years, mean (SD)	Derivation cohort (*n* = 2,369)	External cohort (Ext_A) (*n* = 350)	External cohort (Ext_B) (*n* = 329)
	72.9 (13.5)	71.8 (13.3)	71.3 (12.4)
Male (%)	1,340 (56.6)	193 (55.1)	200 (60.8)
Core volume in mL, median (IQR)	19.7 (9.4–54.0)	21.6 (9.7–55.5)	18.5 (8.9–49.2)
Core volume > 30 mL, number (%)	936 (39.51)	140 (40.0)	127 (38.6)
Labels			
Diffusion-FLAIR match (%)	1,443 (60.9)	213 (60.8)	160 (48.6)
Diffusion-FLAIR mismatch (%)	289 (12.2)	23 (6.6)	69 (21.0)
Subtle FLAIR change (%)	410 (17.3)	63 (18.0)	54 (16.4)
Regional heterogeneous FLAIR change (%)	227 (9.6)	51 (14.6)	46 (14.0)

Summary of demographic characteristics, infarct core volume, and ground truth label distributions in the derivation cohort and two external validation cohorts (Ext_A and Ext_B). Core volume was calculated using an automated segmentation model^16^. The DWI-FLAIR mismatch classification was determined through expert manual labeling based on imaging appearances. Percentages for label categories are calculated relative to the total number of included patients per cohort.

**Fig. 1:**
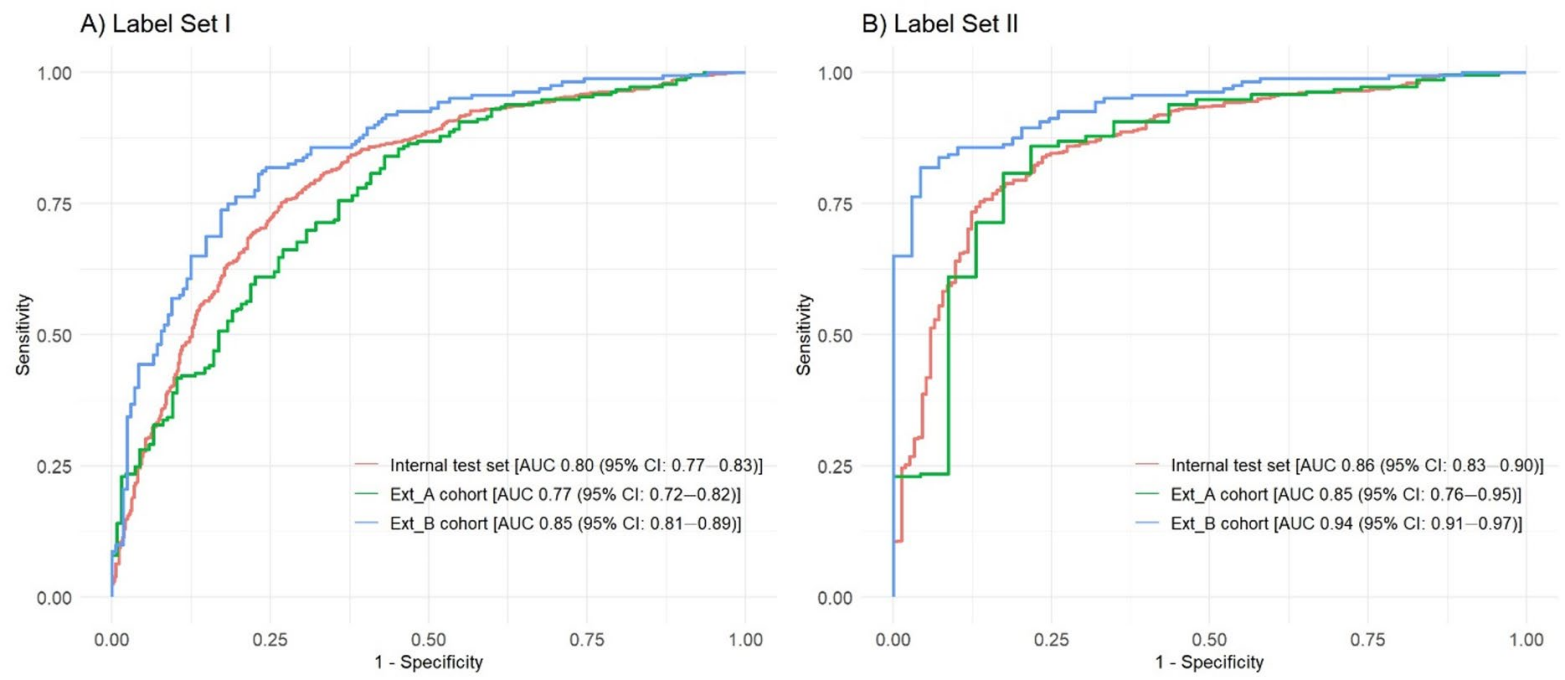
AI Model Performance for DFM Prediction Using B1000 and ADC. Discriminative performance of the deep learning model in predicting diffusion-FLAIR mismatch (DFM) is shown for two label schemes: (A) Broad classification (Label Set I), in which both mismatch cases and those with ambiguous FLAIR findings (such as subtle or regionally heterogeneous changes) were grouped as non-match; and (B) Focused classification (Label Set II), which includes only clearly defined match and mismatch cases. Each plot presents results for the internal test set (red), the external Ext_A cohort (green), and the Ext_B cohort (blue). Area under the receiver operating characteristic (AUROC) curve values with 95% confidence intervals are shown. The model consistently demonstrated improved classification performance under the Focused classification setting across all cohorts.

For the Broad classification, the model achieved an AUC of 0.80 (95% CI: 0.77–0.83) on the internal test set. On the external validation sets, the AUCs were 0.85 (95% CI: 0.81–0.89) for Ext_B and 0.77 (95% CI: 0.72–0.82) for Ext_A. For the Focused classification, the internal test set yielded an AUC of 0.86 (95% CI: 0.83–0.90), while the AUCs for the Ext_B and Ext_A cohorts were 0.94 (95% CI: 0.91–0.97) and 0.85 (95% CI: 0.76–0.95), respectively (Fig. 1). As shown in Table 1, the Ext_B cohort had a significantly higher proportion of clear mismatch cases (21.0%) compared to the Ext_A cohort (6.6%), which may account for the higher classification performance observed in the Ext_B cohort. The calibration slope demonstrated good alignment with observed outcomes (Figure S5), and Brier scores were 0.15 for the internal test set, 0.16 for Ext_A, and 0.23 for Ext_B, suggesting acceptable predictive performance.

Grad-CAM visualizations revealed the specific regions the model attended to during classification. In DF match cases, the model sharply highlighted the infarcted regions, whereas in DF mismatch cases, the attention maps were more diffusely distributed, sometimes extending beyond the lesion boundaries (Figure S6).

**Fig. 2:**
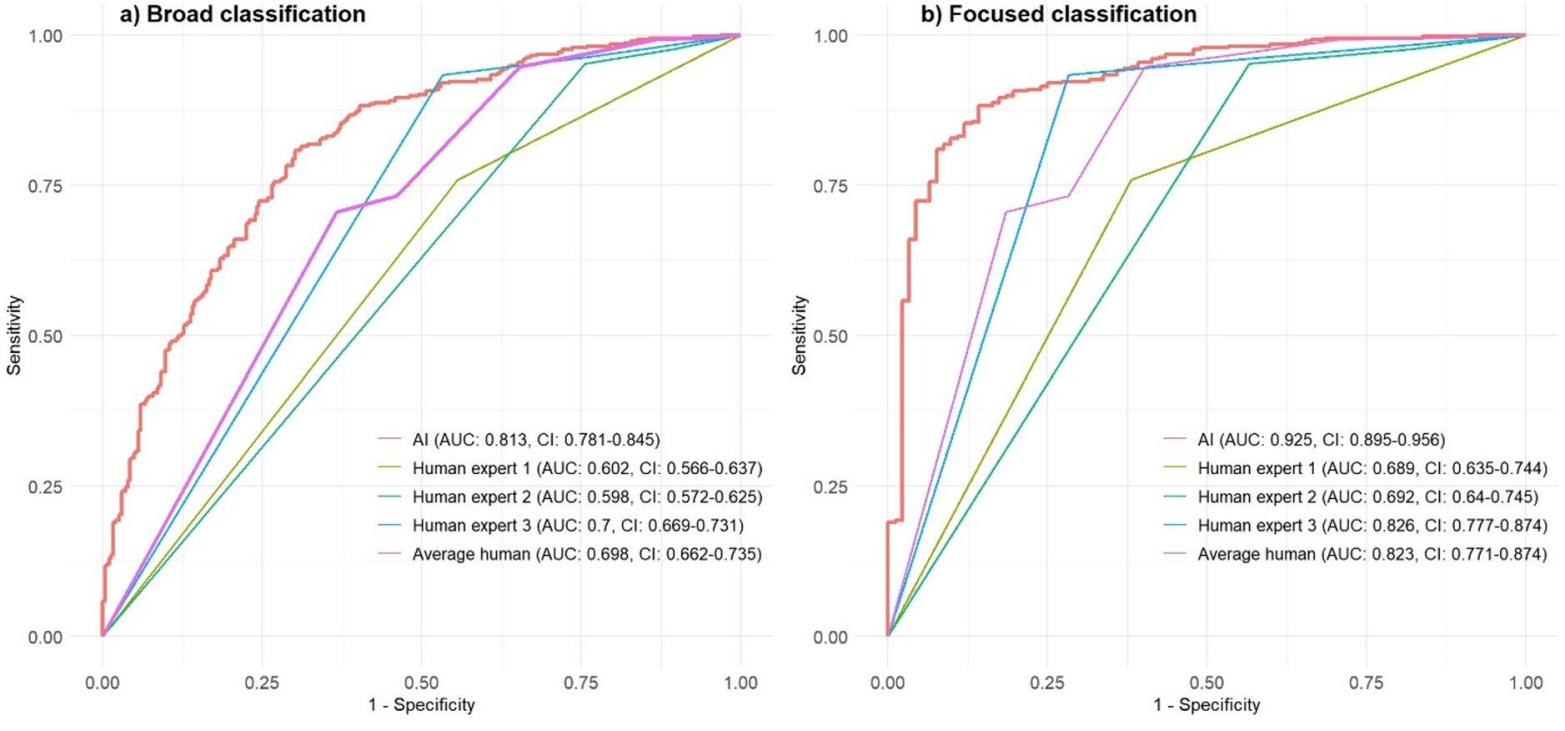
AI vs. Human ROC Curves in Broad and Focused Classification. Receiver operating characteristic curves comparing the diagnostic performance of the AI model and three individual human experts in predicting diffusion-FLAIR mismatch. Each curve corresponds to the AI model (red), three human experts (olive, green, and blue), and their average (magenta). The AUC and 95% confidence intervals are provided in the figure legend. The AI model consistently outperformed individual human experts and the reader average, with the performance gap more pronounced in the Focused classification setting.

In the external validation dataset, under the Focused classification, the AI model significantly outperformed human experts, achieving an AUROC of 0.92 (95% CI: 0.88–0.95) compared to the average human AUROC of 0.82 (95% CI: 0.77–0.87), with a performance difference of 0.10 (95% CI: 0.05–0.16, _p_ < 0.001). In subgroup analyses stratified by clinical and imaging factors, the AI model consistently outperformed human readers (Table 2; Fig. 2). Both the AI model and human experts benefited from the Focused classification, showing improved performance over the Broad classification. In the external validation dataset as a whole, the AUROC of the AI model increased from 0.81 to 0.92 (difference: 0.112, 95% CI: 0.068–0.153, _p_ < 0.001), and that of human experts increased from 0.70 to 0.82 (difference: 0.125, 95% CI: 0.064–0.188, _p_ < 0.001).

**Table 2:** Performance Comparison Between the AI Model and Human Experts on the External Dataset.

	AUROC							
	AI model	Reader 1	Reader 2	Reader 3	Readers (average)	Difference in AUROC		p value^†^
	AI vs. readers (average)							
**Overall dataset** ^*^								
Broad classification (Label Set I, *n* = 679)	0.81 (0.78–0.85)	0.60 (0.57–0.64)	0.60 (0.57–0.63)	0.70 (0.67–0.73)	0.70 (0.66–0.74)	0.11 (0.08–0.16)		< 0.001
Focused classification (Label Set II, *n* = 465)	0.92 (0.88–0.95)	0.69 (0.64–0.74)	0.69 (0.64–0.75)	0.83 (0.78–0.87)	0.82 (0.77–0.87)	0.10 (0.05–0.16)		< 0.001
**Core volume**								
> 30 mL (*n* = 267)	0.83 (0.77–0.88)	0.63 (0.58–0.68)	0.58 (0.54–0.62)	0.73 (0.68–0.78)	0.73 (0.68–0.79)	0.09 (0.02–0.16)		0.006
5–30 mL (*n* = 412)	0.80 (0.76–0.85)	0.58 (0.54–0.63)	0.61 (0.57–0.64)	0.68 (0.64–0.72)	0.68 (0.63–0.73)	0.13 (0.07–0.18)		< 0.001
**Stroke onset time** ^‡^								
< 6 h (*n* = 144)	0.82 (0.72–0.92)	0.63 (0.52–0.73)	0.61 (0.52–0.71)	0.78 (0.70–0.86)	0.74 (0.64–0.83)	0.09 (−0.02–0.2)		0.102
6–24 h (*n* = 237)	0.77 (0.71–0.83)	0.58 (0.52–0.63)	0.58 (0.54–0.62)	0.66 (0.60–0.71)	0.67 (0.61–0.73)	0.1 (0.03–0.17)		0.008
24 h – 1week (*n* = 265)	0.72 (0.64–0.80)	0.54 (0.47–0.61)	0.51 (0.47–0.54)	0.55 (0.50–0.60)	0.55 (0.48–0.63)	0.17 (0.06–0.26)		< 0.001
> 1week (*n* = 5)	0.59 (0.29–0.89)	0.59 (0.34–0.85)	0.5 (0.5–0.5)	0.48 (0.47–0.52)	0.58 (0.31–0.84)	0.01 (−0.3–0.5)		0.945
**Ext_A cohort**								
Broad classification (Label Set I, *n* = 350)	0.77 (0.72–0.82)	0.57 (0.52–0.62)	0.59 (0.55–0.63)	0.65 (0.61–0.70)	0.67 (0.62–0.73)	0.1 (0.03–0.16)		0.002
Focused classification (Label Set II, *n* = 236)	0.85 (0.76–0.95)	0.65 (0.54–0.75)	0.67 (0.56–0.77)	0.74 (0.64–0.85)	0.77 (0.66–0.88)	0.08 (−0.03-0.21)		0.158
**Ext_B cohort**								
Broad classification (Label Set I, *n* = 329)	0.85 (0.81–0.89)	0.63 (0.59–0.68)	0.61 (0.58–0.65)	0.74 (0.70–0.78)	0.73 (0.68–0.78)	0.12 (0.07–0.18)		< 0.001
Focused classification (Label Set II, *n* = 229)	0.94 (0.91–0.97)	0.72 (0.65–0.78)	0.71 (0.65–0.77)	0.86 (0.81–0.92)	0.85 (0.80–0.91)	0.08 (0.03–0.15)		0.004

AUROC values of the AI model and three independent human readers are presented across various subgroups of the external dataset. The “Readers (average)” column indicates the mean AUROC across the three human readers. Subgroup analyses were performed by infarct core volume, stroke onset-to-imaging interval, and individual cohort site (Ext_A and Ext_B). The table also reports the AUROC difference between the AI model and the average of human experts, along with the corresponding p-values.

^*^ The overall dataset refers to the combined external cohorts from Ext_A and Ext_B.

^†^ P-values represent the comparison between the AI model and the average performance of the three human readers, not individual reader comparisons. For core volume subgroup comparisons, DeLong’s test was used. For stroke onset time subgroups, differences in AUROC between the AI model and the readers’ average across multiple time intervals were assessed using a weighted chi-square test, with weights inversely proportional to the variance of each AUROC. All tests were two-sided, and *p* < 0.05 was considered statistically significant. The > 1 week group in the Focused classification was excluded due to 100% match rate.

^‡^ Stroke onset time refers to the interval from symptom onset or last known normal to imaging. Cases with missing onset time (5 in Broad classification, 4 in Focused classification) were excluded from this analysis.

When comparing prediction accuracy between the Broad and Focused classification schemes, both the AI model and human readers exhibited lower performance under the Broad classification. This decline in accuracy was particularly pronounced in cases labeled as non-match that included ambiguous FLAIR features. These findings suggest that ambiguous imaging characteristics contribute to decreased classification reliability for both AI and human experts. (Table 3).

**Table 3:** Comparison of prediction accuracy between the AI model a55388nd human experts

	AI^*^, n (%)	Human experts^†^, n (%)	Both accurate, n (%)	Both inaccurate, n (%)	p value^‡^	p value^§^
Broad classification						
Match (n=373)	301 (80.7)	353 (94.6)	293 (78.6)	12 (3.2)	<0.001	<0.001
Non-match (n=306)	214 (69.9)	106 (34.6)	93 (30.4)	79 (25.8)	<0.001	
Focused classification						
Match (n=373)	329 (88.2)	353 (94.7)	317 (85.0)	8 (2.14)	0.002	<0.001
Mismatch (n=92)	79 (85.9)	55 (59.8)	52 (56.5)	10 (10.9)	<0.001	

## Discussions

We developed a deep learning model capable of classifying diffusion-FLAIR mismatch (DFM) using only diffusion-weighted imaging (B1000) and apparent diffusion coefficient (ADC) images, without the need for FLAIR. The model was validated across multiple external datasets and clinical subgroups, consistently outperforming human experts. These findings demonstrate the feasibility of reliably predicting DFM without FLAIR, offering a clinically practical alternative in acute stroke evaluation.

DFM has emerged as a key imaging biomarker for guiding intravenous thrombolysis in patients with acute ischemic stroke of unknown onset, particularly those with wake-up strokes. According to the WAKE-UP trial^3^,the presence of a DWI lesion without a corresponding FLAIR hyperintensity suggests stroke onset within 4.5 h, identifying patients who may benefit from reperfusion therapy. However, as discussed in the Introduction, FLAIR is not always reliably available or diagnostically usable in real-world acute stroke workflows, which may prevent timely DFM assessment^22^.

To address these limitations, our model was trained to infer the presence of FLAIR signal indirectly by learning subtle imaging features in DWI intensity, texture, and ADC values—patterns that may not be easily discernible to human readers. Previous studies have reported that DWI lesion intensity is associated with the evolution of FLAIR changes^13^, supporting the biological plausibility of this approach. Notably, because B1000 and ADC images are routinely acquired in most acute stroke MRI protocols, our method provides a feasible solution for assessing DFM without requiring additional imaging sequences. By enabling DFM prediction based solely on widely available sequences, this approach offers a practical fallback when standard FLAIR-based assessment is not feasible, potentially broadening the clinical applicability of mismatch-guided treatment decisions.

In this study, the developed model showed robust performance across various conditions, including lesion size, MR vendor, imaging parameters, and magnetic field strength, suggesting its potential for broad applicability and generalizability. However, performance differences were observed in specific patient subgroups and classification schemes. First, the model performed better on the Focused classification set (Label Set II), which excluded ambiguous cases, than on the Broad classification set (Label Set I), which included lesions with subtle or heterogeneous FLAIR changes. In addition, the model’s prediction accuracy tended to decrease in cases with longer onset-to-imaging intervals. This may reflect the progressive attenuation of distinct DFM patterns on DWI and ADC over time, making accurate classification more challenging in delayed imaging scenarios.

Interestingly, the performance decrease in ambiguous cases was observed in both the AI model and human experts, suggesting that the Broad classification captured intrinsic uncertainty in DFM interpretation rather than a model-specific failure. When ground truth labels are poorly defined, AI models struggle to learn consistent patterns, and even experienced experts may find it difficult to make reliable judgments. This highlights the importance of clear and consistent annotation in achieving high diagnostic accuracy.

For similar reasons, lesions smaller than 5 mL were excluded from this study, as such cases often present as multiple scattered, dot-like infarcts for which reliable DFM labeling is inherently difficult. Moreover, these small lesions are rarely relevant to reperfusion decision-making, and their inclusion would risk introducing labeling noise without meaningful clinical gain.

These observations regarding annotation ambiguity and label-dependent performance underscore a long-standing challenge in medical AI: the definition and reliability of ground truth labels. Unlike other domains, raw imaging data in medicine cannot be used directly for supervised learning without expert interpretation. Consequently, if ground truth definitions are inconsistent or vague, the resulting model is prone to error^23,]^^24^. Inter-observer variability and unclear labeling criteria are known sources of performance degradation in both AI and human assessments^25^^[,26^.

To mitigate this, we developed a refined labeling protocol through iterative discussions and consensus review to ensure annotation consistency. Nonetheless, to account for potential inaccuracies in labeling, we designed our experiment to evaluate the impact of label clarity by comparing performance between the Focused and Broad classification sets. The Focused set, which excluded ambiguous cases, consistently resulted in higher model and reader performance. This clearly indicates that both AI and human accuracy decline when ground truth is uncertain. This phenomenon was also reflected in the performance differences between the external validation cohorts.

For example, the model performed better in Ext_B than in Ext_A, which may reflect differences in case mix and class separability. As detailed in Table 1, Ext_B contained a higher proportion of clear mismatch cases than Ext_A, whereas the lower proportion of clear mismatch cases in Ext_A may have reduced class separability and contributed to lower observed performance.

Lastly, it is important to acknowledge that the labels used in this study do not necessarily represent a definitive pathophysiological ground truth. This concern has also been raised in previous studies^27^. Although DWI-FLAIR mismatch has been proposed as an imaging marker of salvageable brain tissue, its definition varies across studies, and a universally accepted clinical interpretation is still lacking. Therefore, before deploying models trained on specific label definitions in clinical practice, further clinical validation studies—based on these same label criteria—are essential to ensure the relevance and reliability of model-driven decisions.

It should be noted that the human–AI comparison in this study was not designed to simulate a realistic clinical scenario in which humans assess DFM without FLAIR. Rather, it was intended as an experimental framework to contrast the capacity of humans and AI to capture mismatch-related latent information embedded in B1000 and ADC images. In this controlled B1000/ADC-only setting, the AI model consistently outperformed human experts across various subgroup analyses, consistent with prior reports of deep-learning systems matching or exceeding clinician performance in acute neuroimaging triage^28^.

Post-study interviews provided additional insight into how readers approached this task. The neuroradiologists reported relying mainly on signal-intensity patterns on B1000 and ADC that they considered suggestive of DWI–FLAIR discrepancy. In contrast, the neurointerventionist reported considering broader imaging cues with clinical relevance, including edema, lesion distribution, vascular territory, and involvement of motor eloquent areas (Table S1). The higher prediction accuracy of the neurointerventionist suggests that DFM inference from B1000 and ADC may depend not only on signal-intensity differences but also on spatial and contextual imaging patterns relevant to treatment-oriented interpretation. This observation may also help interpret the behavior of the AI model. During ground-truth construction, ambiguous cases such as subtle or regionally heterogeneous FLAIR changes were adjudicated not only by visual signal appearance but also by their potential relevance to reperfusion decision-making; for example, lesions with mismatch in motor eloquent areas were classified as non-match. Therefore, the reference labels used for model training may have partly reflected clinically informed imaging interpretation rather than purely signal-based categorization. The AI model may consequently have learned complex imaging patterns aligned with these predefined label criteria. However, this interpretation remains hypothesis-generating, and the model should be regarded as a decision-support tool rather than as a substitute for clinical judgment.

The biological plausibility of this approach can be understood in the context of the pathophysiological and imaging evolution of acute ischemic stroke. In the hyperacute phase, cytotoxic edema typically appears first and is reflected by restricted diffusion on DWI and ADC. As ischemic injury evolves and blood–brain barrier disruption develops, vasogenic edema may follow, prolonging T2 relaxation time and producing hyperintensity on FLAIR images^29^. A DWI–FLAIR mismatch, in which diffusion restriction is present without corresponding FLAIR hyperintensity, is therefore generally interpreted as reflecting an earlier stage of ischemic tissue evolution.

As noted in the Introduction, B1000 images reflect both diffusion restriction and T2-weighted signal components^8,30^, whereas ADC maps provide a more direct measure of diffusion restriction. The combined interpretation of B1000 and ADC may therefore contain indirect information about whether diffusion restriction is accompanied by T2/FLAIR-related tissue changes. In this context, the model’s performance may reflect its ability to integrate relative signal intensity, lesion texture, and spatial distribution patterns across B1000 and ADC images. These findings support the possibility that B1000 and ADC contain useful surrogate information for estimating FLAIR-defined DFM, although this inference remains indirect and should not be interpreted as a direct replacement for FLAIR imaging.

From a clinical perspective, the proposed model should be regarded as a decision-support tool rather than a stand-alone determinant of treatment eligibility. This cautious positioning is particularly important because DWI–FLAIR mismatch itself does not constitute a definitive pathophysiological ground truth, and uncertainties remain regarding its definition and clinical interpretation^27^. Accordingly, the model output should be interpreted as supplementary information within the broader clinical and imaging context, particularly when FLAIR is unavailable, unreliable, or intentionally omitted.

Several features may facilitate practical implementation. First, the model provides continuous probability scores rather than only binary labels, allowing clinicians to distinguish high-confidence predictions from borderline cases. Second, the probability cutoff can be adjusted to prioritize sensitivity or specificity according to local clinical needs, rather than imposing a single fixed decision rule. Third, integration into existing acute-stroke imaging workflows, such as PACS viewers or MRI-based triage dashboards, could allow the model output to be reviewed together with conventional imaging and clinical information. Finally, Grad-CAM–based saliency maps may improve transparency and user familiarity, although they should be interpreted as complementary visual references rather than deterministic spatial explanations.

The diffusely distributed Grad-CAM activation patterns observed in mismatch cases may reflect the nature of the underlying imaging signal. Unlike match cases, in which established vasogenic edema may produce more localized signal changes, mismatch cases are characterized by the relative absence of FLAIR-related tissue changes. Such cases may therefore be better represented by global or distributed signal patterns across B1000 and ADC rather than by a single focal imaging feature.

This study has several limitations. First, the study was based on retrospective data and did not evaluate the impact of the model’s predictions on actual clinical decision-making or patient outcomes. To establish true clinical utility, prospective clinical trials are necessary for further validation.

Second, although the model demonstrated consistent performance across cohorts with varying lesion sizes, MRI vendors, and imaging parameters, its generalizability to all possible MRI configurations remains uncertain. For example, performance in ultra-high field MRI, the latest vendor-specific systems, or unstandardized clinical environments was not assessed. Future studies should evaluate reproducibility and external validity across a broader range of imaging conditions.

Third, the absence of a universally accepted definition of DFM presents a fundamental challenge for both clinical application and model-based prediction, as discussed above. Future efforts should aim to develop novel imaging biomarkers, potentially through deep learning approaches, that can predict salvageable brain tissue more reliably using accessible modalities or multimodal image integration.

In conclusion, we developed a deep learning-based classifier for estimating DFM using only B1000 and ADC images, without FLAIR, and evaluated its performance through external validation across multiple institutions. Within the predefined label schemes used in this study, the AI model showed higher diagnostic performance than human experts. The model may serve as a complementary decision-support tool in acute stroke settings where FLAIR imaging is unavailable, unreliable, or intentionally omitted, supporting—rather than replacing—clinician judgment when considering mismatch-guided treatment eligibility. However, because DFM labeling remains inherently subjective and does not constitute a definitive pathophysiological ground truth, model outputs should be interpreted cautiously and in conjunction with conventional imaging findings and clinical context. Future studies should pursue prospective clinical validation, ideally with patient-outcome endpoints, and evaluate the model across diverse hospital environments to confirm its generalizability and clarify its clinical utility. Multimodal approaches that integrate clinical and imaging data also warrant further investigation.

## Supplementary Information

Below is the link to the electronic supplementary material.

Supplementary Material 1

## Data Availability

Data generated or analyzed during the study are available from the corresponding author by request.
