# Supplementary material for: Predicting diffusion-FLAIR mismatch from B1000 and ADC without FLAIR: A deep learning-based approach
Source: Sci Rep. 2026 Jun 2;16:25084. doi: 10.1038/s41598-026-55388-x (PMC13462109; doi:10.1038/s41598-026-55388-x)
Supplement: Supplementary file 1 [file 41598_2026_55388_MOESM1_ESM.docx]

SUPPLEMENTARY MATERIALS

# Figure S1. Ground Truth Lable Categories for Diffusion-FLAIR Mismatch Classification

Representative MRI scans illustrating the five categorization classes used by expert raters for manual labeling of DWI-FLAIR mismatch (DFM). Each row presents three columns: DWI with infarct mask overlay (left), B1000 image (middle), and FLAIR image (right). (a) Diffusion-FLAIR match: diffusion restriction on B1000 corresponds with FLAIR hyperintensity in the same region. (b) Diffusion-FLAIR mismatch: high signal on B1000 without corresponding FLAIR hyperintensity, suggesting early ischemia. (c) Subtle FLAIR change: mild or faint FLAIR signal change within the diffusion-restricted area. (d) Regional heterogeneous FLAIR change: patchy or mixed FLAIR enhancement within the lesion. In cases involving motor-eloquent areas, regions were labeled as non-match if mismatch features predominated. White arrows indicate matched regions, while arrowheads indicate mismatched areas. (e) Not indicative of acute ischemic stroke or unsuitable for analysis: examples include chronic infarction or hypoxic-ischemic encephalopathy, which were excluded from DFM assessment.
To facilitate binary classification, two labeling strategies were employed. Label Set I (Broad classification) classifies only clearly matched cases (a) as DF match, and groups mismatch (b), subtle (c), and heterogeneous (d) FLAIR cases as non-match. Label Set II (Focused classification) includes only clearly defined match (a) and mismatch (b) categories, excluding ambiguous cases (c and d) to enable clearer diagnostic contrast.

^*^ Images are overlayed with infarct masks predicted using a deep learning–based segmentation model on DWI. The reported volume represents the infarct core size based on the predicted mask.

^†^ DWI refers to diffusion-weighted imaging with b = 1000 s/mm².

Abbreviations: DWI, diffusion-weighted imaging; FLAIR, fluid-attenuated inversion recovery.

# Figure S2. Example Image Set Provided to Human Readers for DFM Assessment

Each case included the B1000 image, the B1000 image with an overlaid infarct mask, and multiple ADC maps with different window settings (default, 1500/500, 1500/1000, and 2500/1000). A total of 36 consecutive axial slices were provided per patient, and readers were allowed to freely explore the entire volume to make an overall judgment regarding the presence of DWI-FLAIR mismatch.

# Figure S3. Five-Fold Cross-Validation Design for Model Development

The derivation dataset consisting of 2,369 patients was divided into five folds for cross-validation. In each fold, approximately 1,895 patients were used for training, and 237 patients each for tuning and testing, ensuring that each sample was included in the test set exactly once. Stratified sampling was applied to maintain consistent label distribution across all splits. Although test sets were mutually exclusive, tuning sets partially overlapped due to the stratification process.
Predicted probabilities from all five test sets were aggregated to form a combined evaluation cohort of 1,185 patients. Model performance metrics were calculated based on this aggregate, allowing a robust estimation of generalizability to unseen data.

# Figure S4. Patient Selection Flow Diagrams for the Derivation and External Validation Datasets A) Derivation data set

B) External validation data set (Ext_A)

C) External validation data set (Ext_B)

Flow diagrams showing the patient selection process for the derivation dataset (A) from two tertiary hospitals, and the external validation datasets from Ext_A (B) and Ext_B (C). Patients with suspected acute ischemic stroke who underwent brain MRI were screened for complete imaging sequences (B0, B1000, ADC, and FLAIR) and lesion volume ≥ 5 cc. Cases meeting these criteria were manually labeled by expert reviewers and included for model training and evaluation based on the classification criteria defined in this study.

_*_ Lesion volume was calculated using a segmentation model.
_†_ Images either did not show evidence of cerebral infarction or were unsuitable for analysis in this study.

# Figure S5. Calibration of the Deep Learning Model in Internal and External Cohorts

Calibration plots comparing predicted probabilities with observed event rates for diffusion-FLAIR mismatch classification. Panels show results for the internal test set (A), external KM cohort (Ext_A) (B), and external YN cohort (Ext_B) (C). A perfectly calibrated model would have predictions that align closely with the diagonal reference line. Each point represents the average observed outcome within a bin of predicted probabilities. Although the Ext_A and Ext_B cohorts show some fluctuations—likely due to smaller sample sizes—overall the model demonstrates consistent agreement between predicted and observed probabilities. The internal test set shows relatively stable and well-aligned calibration performance.

# Figure S6. Guided Grad-CAM Visualization Examples for DF Match and Mismatch Cases

Representative examples of diffusion-FLAIR (DF) match (left) and mismatch (right) cases. Each case includes diffusion-weighted imaging (DWI), FLAIR, apparent diffusion coefficient (ADC), and the corresponding Guided Grad-CAM overlay highlighting regions that contributed to the model's prediction. In the DF match case, high signal intensity is observed on both DWI and FLAIR (white arrows), and the model’s attention is concentrated on the lesion area. In the DF mismatch case, DWI shows high signal without a corresponding FLAIR hyperintensity, and the model’s attention is more diffusely distributed across a broader region. These visualizations suggest that the model may incorporate a wider range of image features when predicting mismatch. However, it remains unclear whether the broader attention reflects uncertainty or is part of the model’s decision-making strategy.

**Table S1. Criteria Used by Human Readers for DFM Classification in the Reader Study**

| **Category** | **Neuro-radiologists** | **Neuro-interventionist** |
| --- | --- | --- |
| DF match | - ‘Marked’ high signal intensity (HSI) in b1000  - Peripheral edema HSI in ADC  - Sulcal effacement / gyral swelling | - Relative iso-signal intensity in ADC  - Multiple scattered lesion |
| DF mismatch | - Differentiated GM/WM in b1000  - Parenchymal normal SI in b1000 | - ‘Marked’ low signal intensity (LSI) in ADC  - Not remarkable HSI in b1000  - No edema in b1000  - Single and large territorial lesion  - Lesion in motor cortex (eloquent area) |

This table summarizes the qualitative criteria used by a neuroradiologist and a neurointerventionist to classify diffusion-FLAIR (DF) match and mismatch cases during the reader study. Although both readers evaluated the same cases, their decision-making processes relied on different visual and clinical cues. The neuroradiologist primarily focused on signal intensity patterns, edema, and anatomical changes such as sulcal effacement or gyral swelling. In contrast, the neurointerventionist incorporated contextual clinical features, including lesion distribution, eloquence of brain regions, and tissue appearance on ADC. These differences in criteria may reflect the distinct clinical priorities of each specialty and contribute to inter-reader variability in DFM interpretation.
